# How does playing adapted sports affect quality of life of people with mobility limitations? Results from a mixed-method sequential explanatory study

**DOI:** 10.1186/s12955-017-0597-9

**Published:** 2017-01-25

**Authors:** Félix Côté-Leclerc, Gabrielle Boileau Duchesne, Patrick Bolduc, Amélie Gélinas-Lafrenière, Corinne Santerre, Johanne Desrosiers, Mélanie Levasseur

**Affiliations:** 1 School of Rehabilitation, Faculty of Medicine and Health Sciences, Université de Sherbrooke, 3001 12th Avenue North, Sherbrooke, QC Canada; 2 Research Center on Aging, Centre intégré universitaire de santé et de services sociaux (CIUSSS) de l’Estrie, Sherbrooke, QC Canada

**Keywords:** Quality of Life Index, Parasports, Well-being, Life satisfaction, Sense of belonging, Wheelchair users

## Abstract

**Background:**

Occupations, including physical activity, are a strong determinant of health. However, mobility limitations can restrict opportunities to perform these occupations, which may affect quality of life. Some people will turn to adapted sports to meet their need to be involved in occupations. Little is known, however, about how participation in adapted sports affects the quality of life of people with mobility limitations. This study thus aimed to explore the influence of adapted sports on quality of life in adult wheelchair users.

**Methods:**

A mixed-method sequential explanatory design was used, including a quantitative and a qualitative component with a clinical research design. A total of 34 wheelchair users aged 18 to 62, who regularly played adapted sports, completed the Quality of Life Index (/30). Their scores were compared to those obtained by people of similar age without limitations (general population). Ten of the wheelchair users also participated in individual semi-structured interviews exploring their perceptions regarding how sports-related experiences affected their quality of life.

**Results:**

The participants were 9 women and 25 men with paraplegia, the majority of whom worked and played an individual adapted sport (athletics, tennis or rugby) at the international or national level. People with mobility limitations who participated in adapted sports had a quality of life comparable to the group without limitations (21.9 ± 3.3 vs 22.3 ± 2.9 respectively), except for poorer family-related quality of life (21.0 ± 5.3 vs 24.1 ± 4.9 respectively). Based on the interviews, participants reported that the positive effect of adapted sports on the quality of life of people with mobility limitations operates mainly through the following: personal factors (behavior-related abilities and health), social participation (in general and through interpersonal relationships), and environmental factors (society’s perceptions and support from the environment). Some contextual factors, such as resources and the accessibility of organizations and training facilities, are important and contributed indirectly to quality of life. Negative aspects, such as performance-related stress and injury, also have an effect.

**Conclusions:**

People with mobility limitations playing adapted sports and people without limitations have a similar quality of life. Participation in adapted sports was identified as having positive effects on self-esteem, self-efficacy, sense of belonging, participation in meaningful activities, society’s attitude towards people with mobility limitations, and physical well-being. However, participants stated that this involvement, especially at higher levels, had a negative impact on their social life.

## Background

In Canada, nearly two million people live with mobility limitations [1]. Many of them need to use wheelchairs to get around, which can limit the activities available to them [2], leading to restrictions in their social participation and reduced quality of life. Quality of life reflects individuals’ cognitive and emotional reactions to their accomplishments, according to the cultural context and value system in which they live, in relation to their goals, aspirations, standards and concerns [3–5]. Greater congruence between an individual’s aspirations and accomplishments leads to positive reactions, including satisfaction with life, physical, mental, social and spiritual well-being, feeling of control over one’s life, and sense of accomplishment of meaningful occupations [6]. Conversely, a lack of congruence or too great a gap between aspirations and accomplishments can produce negative reactions like dissatisfaction or depression [6]. According to the literature, self-esteem [7], self-efficacy [8, 9] and the sense of belonging to a group [10, 11] are important personal factors to consider in fostering good quality of life. Self-esteem is defined by how good a person feels about him/herself [7]. Self-efficacy reflects the beliefs a person has about his/her abilities and is influenced by events that affect his/her life [8]. It is also a potential factor influencing social participation, which was linked to good quality of life and well-being [9, 10]. Finally, the sense of belonging to a group is the ability to consider oneself and feel like an integral part of a group, family or whole [10].

Quality of life is also associated with participation in meaningful and rewarding occupations such as leisure activities. Leisure is associated with quality of life through its well-documented contribution physically and mentally [12, 13], and leisure activities are known to trigger positive reactions like enjoyment, feeling of well-being, personal satisfaction, self-esteem [14, 15] and a zest for life [13]. Thus, to enable themselves to enjoy satisfactory quality of life, some people with mobility limitations decide to engage in leisure activities adapted to their condition, such as competitive adapted sports [16, 17]. Adapted sports refer to sports modified or created to meet the needs of individuals with disabilities [18]. Playing an adapted sport can be a rewarding experience that creates personal satisfaction [19], good self-esteem [12], and a feeling of proficiency [20]. In addition to enabling them to enjoy the ensuing physical and psychological benefits, playing an adapted sport could make a positive contribution to the quality of life of people with mobility limitations. According to some studies, participating in an adapted sport helps to develop a sense of belonging to a group [20] and, during the rehabilitation phase, makes it easier to accept physical disabilities [20]. Compared to people who do not play a sport, adapted sports help to develop a more positive view of one’s health and a feeling of well-being [12].

Although playing a wheelchair adapted sport has positive psychological and physical effects on people with mobility limitations, little attention has been paid to its impact on their quality of life. Among the few studies done, one involving people with mild cerebral palsy showed that identifying as an athlete affects one’s quality of life [21]. Another found that the quality of life of people with disabilities who played sports was superior to that of people with disabilities who did not [22]. A third study compared spinal cord-injured sport participants and non-participants and found that quality of life and community integration among the sport participants was greater than among the non-participants [23]. To our knowledge, however, no study has compared the self-rated quality of life of people with mobility limitations who play adapted sports and people without disabilities. Thus the objective of this study was to: 1) compare the subjective quality of life of adults with mobility limitations playing a wheelchair adapted sport to that of a population reporting no mobility limitations, and 2) explore the influence of playing an adapted sport on the quality of life of adults with mobility limitations.

## Methods

### Study design

This study employed a mixed-method sequential explanatory design including two components, a quantitative component and a qualitative component with a clinical research design [24–26]. In the quantitative component, the quality of life of adult wheelchair users playing an adapted sport was compared to that of people without disabilities, playing a sport or not. This comparison group was drawn from a convenience sample in the study by Lacroix and colleagues [27], some perfectly matched on age; the widest gap was 13 years and the large majority (94.1%) reported doing physical activities to keep fit. As men outnumbered women in the participants’ group, male participants were matched to comparison group’s women of the control group in 11 instances. The qualitative component involved an in-depth exploration of the influence of playing an adapted sport on quality of life and, more specifically, on self-esteem, self-efficacy and sense of belonging to a group, including the sports community.

### Participants

To be included in the study, participants had to: 1) be between 18 and 64 years of age, 2) use a manual wheelchair every day to get around, 3) have played an adapted sport at least once a week for at least four months, and 4) not present any cognitive or communication problems, according to the student researchers’ clinical judgement. Thirty-four participants met these criteria and were enrolled. There were 9 women and 25 men aged from 18 to 62 years old. To help organize information meetings about the study with members of their team or sports club, the cooperation of contact persons in the Quebec adapted sports network (coaches, sports directors and athletes) was sought. Some participants were also recruited directly during sports competitions.

From the group of 34, a subsample of ten participants, five women and five men, with differing levels of quality of life and different characteristics participated in the qualitative component. More specifically, these participants were selected on the basis of their results on the questionnaire used (Quality of Life Index, see below) in order to have the widest possible variety of experiences represented.

### Data collection

The participants in the quantitative component answered two self-administered questionnaires, one on their personal characteristics and the other on their quality of life (see below). For the qualitative component, individual semi-structured interviews lasting 30 to 60 min were digitally audiotaped, transcribed and verified to ensure the wording used by participants was respected. The interviews were conducted by five student-researchers (in the last year of a four-year master’s program in occupational therapy); each conducted two interviews, at the participants’ homes (*n* = 32) or by phone (*n* = 2). The interviews took place within a 4-month period, and the majority (8; 80%) involved one student-researcher interviewer and another student-researcher observer. The research protocol was approved by the Research Ethics Committee of the Centre intégré universitaire de santé et de services sociaux de l’Estrie – Centre hospitalier universitaire de Sherbrooke (CIUSSS de l’Estrie-CHUS).

### Measuring instruments

#### Quality of life index

The Ferrans and Powers Quality of Life Index (QLI) [28, 29] was used to measure quality of life. The QLI is a well-known and widely-used generic satisfaction with life tool (translated into 9 languages and used in clinical and research settings in 18 countries). It takes the individual’s reactions into account [30], includes norms [29] and has been used with individuals with varying disability levels [31]. The tool consists of two parts, each of which includes 32 items related to four life domains: 1) health and functioning, 2) socioeconomic, 3) psychological/spiritual, and 4) family. The first part of the questionnaire concerns the person’s satisfaction with items in each of the domains (6-point Likert scale ranging from ‘very dissatisfied’ to ‘very satisfied’), while the second part estimates the importance to the respondent of each item (from ‘very unimportant’ to ‘very important’). The total score and score for each of the four domains ranges from 0 to 30. To obtain these scores, 3.5 units are subtracted from each satisfaction item, and the result is multiplied by the level of importance associated with that item, providing a weighted score for each item. The mean scores for the weighted items are then calculated, and 15 is added to eliminate any negative scores [28, 29].

The QLI presents good concurrent validity with satisfaction with life in general (r = 0.65 to 0.75), good test-retest reliability (r = 0.81 in a group of students and 0.87 in a group of dialysis patients) and high internal consistency (Cronbach’s alphas of 0.90 and 0.93, respectively, in the aforementioned groups) [28, 29]. According to the study by Ferrans and Powers [28], the total mean score for the general population (mean age 48.4 +/− 16.8 years) is 23 (S.D. = 4.0); a score of 19 or less suggests poor quality of life and a difference of 2 or 3 points is clinically significant.

#### Sociodemographic and clinical questionnaire

A self-administered questionnaire covering sociodemographic and clinical characteristics was used to collect data on the participants (see Table 1).

**Table 1:** Participants’ sociodemographic and clinical characteristics

	Quantitative component (*n* = 34)	Qualitative component (*n* = 10)
Continuous variables [mean (S.D.)]		
Age (years)	37.7 (9.9)	39.2 (11.1)
Schooling (years)	13.8 (3.1)^a^	14.2 (2.0)
Number of years playing the main sport	8.1 (7)	9.4 (8.7)
Categorical variables [frequency (%)]		
Sex (men)	25 (73.5)	5 (50)
Main language (French)	33 (97.1)	10 (100)
Main diagnosis		
Paraplegia	18 (52.9)	6 (60)
Tetraplegia	7 (20.5)	2 (20)
Amputation	2 (5.9)	
Cancer	2 (5.9)	1 (10)
Other	5 (14.8)	1 (10)
Marital status		
Single	17 (50.0)	5 (50)
Common-law/married	17 (50.0)	5 (50)
Main sport		
Athletics	8 (23.6)	5 (50)
Adapted tennis	8 (23.6)	1 (10)
Rugby	6 (17.6)	1 (10)
Paracycling	5 (14.7)	1 (10)
Basketball	3 (8.8)	1 (10)
Other	4 (11.8)	1 (10)
Level of competition of the sport		
International	11 (32.4)	3 (30)
National	11 (32.4)	4 (40)
Provincial	3 (8.7)	
Other (recreational)	9 (26.5)	3 (30)
Main occupation		
Working	15 (44.1)	1 (10)
Receiving disability benefits	13 (38.2)	5 (50)
Funded athlete	4 (11.8)	2 (20)
Student	2 (5.9)	2 (20)

^a^ *n* = 33

#### Interview guide (qualitative component)

Prior to data collection, a semi-structured interview guide was developed for quality of life and associated dimensions identified in the literature and related to the quality of life theoretical model [4], namely self-esteem, self-efficacy and sense of belonging to a group. Examples of questions included: “*How does playing your adapted sport affect your quality of life?*” or “*Tell me about your social life related to playing your sport*”. This guide was verified during the first interview and modified and expanded based on the participants’ answers. Each student-researcher was trained by an experienced qualitative researcher to conduct the interviews and practised with a participant from the quantitative part of the study who was not selected for the qualitative interview. These practices were recorded, commented on by experienced qualitative researchers and shared with the other interviewers as a training method to ensure uniformity in the process and greater efficacy of the interviews.

### Data analysis and sample sizes

#### Quantitative component

The participants’ characteristics were first described by mean and standard deviation for continuous variables, and frequency and percentage for categorical variables. Using a *t*-test for independent groups, performed with SPSS, results on the QLI obtained by participants with mobility limitations were compared with the QLI scores of 34 people of comparable age, drawn from Lacroix et al. [27] and obtained from the general population, i.e., people between 18 and 64 years of age without any mobility problems (comparison group). With a standard deviation of 5 [standardized mean difference (effect size) of 3/5 = 0.60], alpha error of 5% and power of 80%, a sample size of 34 participants is sufficient to detect a minimum difference of 3 points on the QLI between these two types of participants [32].

#### Qualitative component

A thematic content analysis was carried out simultaneously with data collection, using a lexical guide, summary sheets and a mixed coding grid [33] to systematically identify [34] and add categories as the analysis proceeded. The themes underlying the 3 general categories in the interview guide (self-esteem, self-efficacy and sense of belonging) emerged from this content analysis. Memos containing thoughts, questions, syntheses and discussions by the research team were also used [33]. After each interview was analyzed by at least two authors to enhance the credibility and confirmability via triangulation, the research team met to discuss the coding and modify the interview guides to allow exploration of emerging themes. The thematic content analysis was conducted using the Human Development Model - Disability Creation Process (HDM-DCP) [35] to identify and synthesize existing themes in a systematic process [34], and the emergence of additional categories based on new items identified during the analysis [36]. The HDM-DCP is an ecosystemic conceptual model illustrating the interaction of personal and environmental factors that result in social participation, i.e. all valued daily activities and social roles [35]. Personal factors include identity characteristics (e.g. age, sex, sociocultural identity, resilience and spirituality), organic systems (e.g. nervous and skeletal systems) and capabilities (e.g. intellectual, behavioral, motor). Environmental factors include social and physical facilitators and obstacles to social participation [35]. The analysis was carried out in Word and achieved theoretical data saturation.

## Results

### Quantitative component

The 34 participants in the quantitative component were between 18 and 62 years of age; the majority were men with paraplegia, French-speaking, working, who played an individual adapted sport (mainly athletics, tennis or rugby) at an international or national competitive level (Table 1).

The total score on the QLI of participants with mobility limitations indicated good quality of life (Table 2). The scores obtained for the four domains of the QLI were similar and did not differ between the participants with mobility limitations and the comparison group, except for the family domain. For this domain, a statistically (*p* = 0.03) and clinically (3 points) significant difference was observed between the groups, with the comparison group scoring higher.

**Table 2:** Comparison of the quality of life of participants with mobility limitations playing an adapted sport to that of people without limitations from the general population

QLI (/30)	Participants with mobility limitations playing an adapted sport (*n* = 34)	Participants without limitations from the general population (*n* = 34)	*P* value
1. Health and functioning	21.9 (4.1)	22.4 (3.2)	0.71
2. Socioeconomic	22.1 (4.2)	21.9 (3.3)	0.70
3. Psychological/spiritual	21.9 (4.8)	21.6 (3.1)	0.71
4. Family	21.0 (5.3)	24.1 (4.9)	0.03
Total score	21.9 (3.3)	22.3 (2.9)	0.64

*QLI* quality of life index

### Qualitative component

**Fig. 1:**
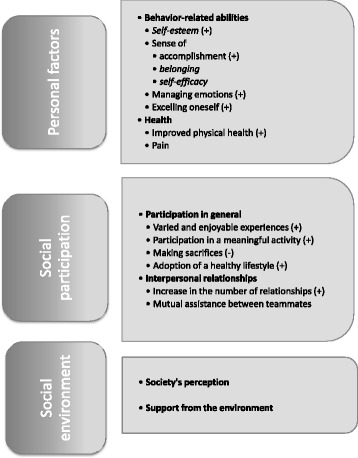
Summary of the effects of sport on quality of life. Themes in italics were specifically mentioned during the interviews. Some themes had a uniquely positive (+) or negative (−) impact on quality of life

Five women and five men, all French-speaking, participated in the qualitative component (Table 1). They mainly had paraplegia, received benefits and were involved in athletics. These participants reported that adapted sports had a direct impact on their quality of life, especially by enhancing their physical well-being and health: “*You are more active, you feel better about yourself, you sleep better.*” (P3). This has a ripple effect on their personal factors, social participation and environment (Fig. 1). The impact was not as great, however, on those participants whose social participation was already good.

#### Personal factors

Playing an adapted sport has a positive impact on physical and psychological factors, especially behavior-related abilities, including self-esteem, self-efficacy and sense of belonging (Fig. 1): “*By getting involved in sport, […] I discovered I had many strengths, lots of things other people don’t have*.” (P2). In addition, the participants said they transferred what they learned, as shown in the following extract concerning an increase in self-efficacy: “*Through sport, I learned I could achieve my objectives, so I apply this in my everyday life*.” (P9). Success in sports also helps them develop a sense of accomplishment, as shown by the following experience in international sports: “*It’s an accomplishment [that] spreads through everyone around you, through your whole life*.” (P5).

In addition, identification with teammates and a sense of fairness foster their feeling of belonging, as indicated by two participants: “*We’re all at the same height [in a wheelchair], we’re all equal*.” (P4) and “*We understand each other, I think that’s an aspect you don’t get [in other social groups]*.” (P2). For one of the female participants, the magnitude of this sense of belonging was considerable: “*[My team] is like my family*.” (P9). Playing an adapted sport also influences quality of life by helping to develop the ability to manage emotions: “*After sports, we try to gain some perspective, find some meaning*.” (P2). Finally, by developing their physical capacities, adapted sports makes their day-to-day activities easier: “*We are stronger physically, transfers are easier*.” (P5). However, playing sports intensively can cause injuries and reduce quality of life: “*You can wear out faster the muscles that need to be functional to help you in your everyday life*.” (P4). Some participants said they experienced stress related to the pressure to perform in competitive sports.

#### Social participation

The influence of playing adapted sports on the participants’ quality of life was also created through social participation, in general and through interpersonal relationships (Fig. 1). Adapted sports provide a variety of enjoyable experiences like travel: “*It [adapted sport] gave me a real social life […] I developed a big social network with friends around the world.*” (P9). Playing an adapted sport also influences quality of life by increasing participation in meaningful activities: “*Since I wasn’t doing any sport, I felt a bit lost because I was no longer doing what I loved.*” (P1). Some competition level participants, however, reported that playing adapted sports involved sacrifices: “*You have to give up certain privileges […] you have to go to bed early instead of going to a party.*” (P2). Finally, playing an adapted sport has a positive impact on quality of life by increasing the number of meaningful relationships with teammates and fostering mutual assistance: “*It’s my group of wheelchair ‘friends’ […] we can help each other.*” (P3).

#### Environment

Playing an adapted sport also influences the participants’ quality of life and self-esteem by changing society’s attitude to people with mobility limitations: “*You have two profiles: you have people in wheelchairs and you have athletes in wheelchairs. As soon as you get active in a sport, people look at you differently*” (P3). Playing adapted sports increases people’s involvement in their community and fosters the opportunity to build a reliable social environment: *“Yes, we talk about sports but he is really a friend. We talk about anything, we see each other, hang around together and it really brings something different”* (P3). Participation in higher levels of competition, however, tends to involve sacrifices in the social environment, particularly for families, which has a negative impact on athletes’ quality of life: “*If you have a family and you’re gone for two months, it’s harder to manage”* (P1). Participation in adapted sports also helps people develop the physical strength to overcome environmental barriers, such as moving in snow: “*You think it’s difficult to roll your chair, then you realize it’s easier than you think*” (P5). Participants also linked playing adapted sports to better physical well-being: “*It gives me a sense of physical wellness and the pleasure of participating in a sport that makes me feel good in my body*.” (P9) When financial, human and physical resources (equipment costs and transportation, coaches, funding for teams) are limited, participation is restricted, which creates dissatfaction with playing sports. Some participants also reported having problems with organizational accessibility (limited number of teams, schedule) and training facilities, which might contribute to reduced quality of life.

## Discussion

The aim of this study was to compare quantitatively the quality of life of people with mobility limitations playing adapted sports to that of a population without disabilities, and to explore the influence of playing adapted sports on the quality of life of participants with mobility limitations. The results show that the quality of life of wheelchair users who play an adapted sport is comparable to that of people without disabilities, and that playing adapted sports influences quality of life through personal factors and social participation of people with mobility limitations. These findings are similar to those in several other studies [9, 22, 23] where sport participants with disabilities had better quality of life and life satisfaction than non-sport participants with disabilities, and community and social participation were linked to good quality of life, which can be expressed by sports participation. The present study shows that adapted sports could impact the quality of life of people with disabilities to a point where it can be compared not only to that of non-sport participants with disabilities but also to that of people without disabilities, whether they are involved in sports or not.

Although some studies showed that using a wheelchair leads to restrictions in social participation [2, 23], which in turn has a negative impact on quality of life [12], the participants in the present study reported an increase in their social participation from playing adapted sports, especially through interpersonal relationships and doing meaningful activities. The results also suggest that an improvement in health-related personal factors, through the development of capacities, helps with the performance of daily activities (Fig. 1). Playing an adapted sport also has a positive impact on self-esteem, self-efficacy and sense of belonging to a group [14, 20, 23, 35, 37, 38]. This effect could explain the similar results for quality of life obtained by the participants with mobility limitations and the comparison group. It is possible that a response shift, i.e., the theory that people may change how they evaluate their quality of life following a trigger event [39], had a positive effect on the participants’ quality of life. By redefining how they rate their quality of life, people with disabilities report high quality of life despite the challenges associated with their reduced mobility.

Quantitative comparisons between our participants and a comparison group from the general population, however, revealed a difference in family-related quality of life, with people with mobility limitations scoring lower than people from the general population. This difference could be attributable to the sacrifices needed to play an adapted sport. Many participants playing at a competitive level reported having difficulty balancing sport and family, which could result in dissatisfaction in family life. One previous study carried out with international athletes with cerebral palsy showed that playing adapted sports had a positive impact on their quality of life in general and their social life, but not on the quality of their family life or family participation [21].

In addition, playing an adapted sport can have a negative impact on some personal factors that can affect quality of life. Participants in the present study reported occasional pain after playing sports. Pain can alter participation in daily activities, as shown in one study in which pain affected adolescents’ participation in daily life and was magnified by age [40]. Stress related to the pressure to perform in competitive sports is another phenomenon that was recognized in a study of high level athletes [41]. Other external factors, including financial, human and physical resources and problems related to organizational accessibility, also affect the playing of adapted sports and are considered to be stressors that can affect sports performance [41]. This stress has negative effects on psychological and physical health [42]. Since these factors can alter participation or affect quality of life, they must be considered when a person with mobility limitations gets involved in playing an adapted sport.

This study has some strengths, including the use of a mixed-method design that enabled us to explore in depth the quality of life of participants with various adapted sports’ backgrounds and triangulate the quantitative and qualitative data. The entire team was involved in the analysis, and the triangulation of the researchers’ perspectives enriched the results. The participants in the qualitative component also had different characteristics, which allowed us to explore a variety of experiences. However, the number of participants in the study was relatively small and they were mostly French-speakers, which might limit the transferability of the results to a particular cultural context. In addition, the pairing with people without disabilities was mainly based on the participants’ age but this led to sex differences in the matched participants. The study was conducted by five occupational therapy students; although they were specially trained and supported by experienced researchers, there may have been some differences in how the qualitative data were collected and analyzed. Finally, as with any study of this type, answers to the questions are subject to social desirability bias, even though the participants were told that there were no right or wrong answers, that it was important to reflect their situation as accurately as possible, and that the data would be kept confidential.

## Conclusions

The results of this study show that the quality of life of people with mobility limitations who play adapted sports is similar to that of the general population, except for family-related quality of life. This similarity may be attributable to the positive impact of adapted sport on personal factors and social participation. Moreover, family-related differences could stem from the sacrifices required to play adapted sports and their effect on family life. The study also showed that some contextual factors, such as resources and the accessibility of organizations and training facilities, are important for playing an adapted sport and contributed indirectly to quality of life. Personal factors (behavior-related abilities and health) and social participation (in general and through interpersonal relationships) also have a direct impact on the quality of life of people with mobility limitations who play adapted sports. Society’s perception and support from the environment also contributed. Some negative aspects, such as performance stress and injuries, also have an effect.

## Abbreviations

CIUSSS de l’Estrie-CHUS: *Centre intégré universitaire de santé et de services sociaux de l’Estrie – Centre hospitalier universitaire de Sherbrooke*
FRQS: *Fonds de la recherche du Québec–Santé*
QLI: Quality of life index
